# Safe use of radiopharmaceuticals in patients with chronic kidney disease: a systematic review

**DOI:** 10.1186/s41181-021-00145-w

**Published:** 2021-08-21

**Authors:** Nanno Schreuder, Iris de Romijn, Pieter L. Jager, Jos G. W. Kosterink, Eugène P. van Puijenbroek

**Affiliations:** 1 Groningen Research Institute of Pharmacy, Unit of PharmacoTherapy, Epidemiology and Economics, University of Groningen, Groningen, The Netherlands; 2 GE Healthcare Radiopharmacy Zwolle, Zwolle, The Netherlands; 3 Division of Pharmacoepidemiology and Clinical Pharmacology, Utrecht Institute for Pharmaceutical Sciences, Utrecht University, Utrecht, The Netherlands; 4 Department of Nuclear Medicine, Isala Hospital, Zwolle, The Netherlands; 5 Department of Clinical Pharmacy and Pharmacology, University Medical Center Groningen, University of Groningen, Groningen, The Netherlands; 6 Netherlands Pharmacovigilance Centre Lareb, ’s-Hertogenbosch, The Netherlands

**Keywords:** Radiopharmaceuticals, Nuclear medicine, Chronic kidney disease, Renal insufficiency, Drug dosage calculations, Drug safety

## Abstract

**Background:**

Patients with chronic kidney disease (CKD) may need to have their radiopharmaceutical dosage adjusted to prevent adverse effects and poor outcomes, but there are few recommendations on radiopharmaceutical dosing for this group of patients. The aim of this study is to provide an overview of the available information on radiopharmaceutical dose recommendations for patients with CKD.

**Methods:**

We performed a systematic literature review according to the Preferred Reporting Items for Systematic Reviews and Meta-Analyses (PRISMA) statement. We conducted a literature search in the MEDLINE (PubMed) and Embase databases and screened potentially relevant studies using inclusion and exclusion criteria. We independently assessed the included observational studies’ methodologies and extracted relevant data.

**Results:**

Of the 5795 studies first identified, 34 were included in this systematic review. These studies described three radiopharmaceuticals: [^131^I]sodium iodine, [^18^F]fludeoxyglucose, and [^131^I]iobenguane. Twenty-nine studies (85.3%) reported data on patients with CKD stage 5, while only three studies mentioned CKD patients in other stages (8.8%).

**Conclusion:**

We found no consistent recommendations for radiopharmaceutical dosing in patients with CKD. Although some studies do mention dosing difficulties in patients with CKD, information is available for only a few radiopharmaceuticals, and recommendations are sometimes contradictory. Further research on radiopharmaceutical dosing in patients with CKD is needed to determine whether these patients require specific dosing, especially for therapeutic radiopharmaceuticals where a non-optimised dose may lead to an increased risk of toxicity for non-targeted organs. Including patients with CKD in studies and providing specific information about dosing in these patients should be a priority for the radiopharmaceutical community.

## Introduction

Nuclear medicine plays an important role in the diagnosis and therapy of diseases, particularly in the field of oncology. The field of nuclear medicine relies on radioactive compounds, so-called radiopharmaceuticals (Smith et al. 2012). Selecting the right dose of a radiopharmaceutical, expressed in becquerels (Bq) as the activity of the compound’s radionuclide, is of high importance. For diagnostic radiopharmaceuticals, the ideal dose will provide accurate, useful diagnostic information while keeping the radiation dose to the patient low (Pickett and Theobald 2011; Fahey and Stabin 2014). The efficacy will depend on the biodistribution of the diagnostic radiopharmaceutical. Important aspects include localisation in a target organ, localisation in non-target organs, and the mechanisms—such as biological excretion—for removing non-target radioactivity. Advantageous biodistribution will contribute to a good target-to-non-target activity ratio, ensuring optimal image quality which allows a clear diagnostic outcome (Pickett and Theobald 2011). For therapeutic radiopharmaceuticals, the ideal dose will deliver the right therapeutic activity without causing adverse effects and with a minimum radiation dose to non-target organs or tissues (Chan et al. 2011; Kassis and Adelstein 2005; Meredith and Wessels 2008). The biodistribution of the therapeutic radiopharmaceutical is important because localisation in the target organ will determine the therapeutic response, and non-target organs are at risk of toxicity (Pickett and Theobald 2011).

Chronic kidney disease (CKD), a growing health problem with an estimated prevalence of 11–13% in the general population, is characterised by kidney function decline. It can result from diseases, such as diabetes mellitus and hypertension, or from aging (Hill et al. 2016; Girndt et al. 2016; Nissenson et al. 2001). CKD may reduce the excretion rate of pharmaceuticals and their metabolites, elevating plasma concentrations and requiring the dose to be adjusted (Matzke and Frye 1997; Dreisbach and Lertora 2008). Similarly, for radiopharmaceuticals that are cleared by the kidneys, the biodistribution of the radioactive drug is likely to be altered in patients with CKD. For diagnostic radiopharmaceuticals, decreased clearance may lead to prolonged blood pool activity and subsequently to a poor target-to-non-target ratio, which may decrease image quality and ultimately affect the diagnostic outcome. For therapeutic radiopharmaceuticals, decreased clearance may lead to increased activity at the target organs or non-target organs, which increases the risk of toxicity. Therefore, it is expected that the radiopharmaceutical dosage in patients with CKD will have to be adjusted (Krens et al. 2019; Munar and Singh 2007).

At present, the dose of a radiopharmaceutical is fixed in most cases, although it is sometimes adjusted for body weight, as in the case of children and obese patients (Sjögreen Gleisner et al. 2017; Lassmann and Treves 2014). However, standards for radiopharmaceutical dosing in patients with CKD are lacking. One review describes treatment with radioiodine for hyperthyroidism and thyroid cancer in end-stage CKD. The review mentions that the available literature is scarce and that standards, based only on analysis of single case reports, are not coherent (Saracyn et al. 2014). Some available nuclear medicine guidelines contain only one paragraph on the use of radiopharmaceuticals in patients with CKD. Only one nuclear medicine guideline gives a specific dose recommendation for these patients, advising that the administered dose of bone-seeking therapeutic radiopharmaceuticals for palliation of bone pain should be lowered by 50% in patients with creatinine clearance of less than 50 mL/min (Bodei et al. 2008a). Other guidelines provide only general, nonspecific comments, such as recommending that renal function should be assessed, that a nephrologist should be consulted, that the administration of the radiopharmaceutical should be carefully planned and managed, or even that patients with CKD should be excluded (Bodei et al. 2008a; Poeppel et al. 2018; Wyngaert et al. 2016; Bombardieri et al. 2010; Silberstein et al. 2012; Zaknun et al. 2013; Hope et al. 2019; Luster et al. 2008). While these effects are particularly relevant in therapeutic applications because a change in biodistribution may affect therapy outcomes or increase the risk of toxicity in these patients, diagnostic radiopharmaceutical guidelines also indicate that scans obtained in CKD patients may be suboptimal due to a change in biodistribution. Some suggest increasing the time between administration of the radiopharmaceutical and imaging (Wyngaert et al. 2016; Boellaard et al. 2015; Bartel et al. 2018; Dam et al. 2014; Practice Guideline 2009). However, several guidelines for both therapeutic and diagnostic radiopharmaceuticals mention that while dose adjustment may be needed in this group of patients, little is known about this topic (Poeppel et al. 2018; Bombardieri et al. 2010; Zaknun et al. 2013; Silberstein et al. 2003; Balon et al. 2011; Djang et al. 2012).

Therefore, our aim in this systematic review is to provide an overview of the available information on radiopharmaceutical dose recommendations for patients with CKD.

## Methods

### Study design

We conducted this systematic literature review according to the Preferred Reporting Items for Systematic Reviews and Meta-Analyses (PRISMA) statement (Moher et al. 2009), and the review was registered in the International Prospective Register of Systematic Reviews (PROSPERO) under number CRD42019136107 (Centre for Reviews and Dissemination 2019).

### Search strategy

We performed a computerised literature search using the databases MEDLINE (PubMed) and Embase. Two researchers (I.d.R. and N.S.) developed a search string for each database (Table 1) using keywords for both CKD and radiopharmaceuticals. No publication year limits were applied. Only studies in the English language were included, and a filter was applied to exclude animal-only studies. An additional filter was applied in Embase to exclude studies available in MEDLINE. We screened the selected studies and review articles to identify additional relevant studies and references. The initial search was completed on 10 May 2019 and updated with recent articles until 7 October 2020.

**Table 1:** Search strategies employed for PubMed and Embase

Database	Search string
PubMed	(((((Radiopharmaceuticals(MeSH) OR radiopharmaceutical*(tiab) OR radioactive drug*(tiab) OR radioiodine(tiab)))) AND ((Kidney Diseases(MeSH) OR kidney disease*(tiab) OR Renal Insufficiency(MeSH) OR renal insufficien*(tiab) OR renal impairment(tiab) OR Glomerular Filtration Rate(Mesh) OR glomerular filtration rate*(tiab) OR eGFR(tiab) OR Metabolic Clearance Rate(MeSH) OR renal clearance(tiab)))) NOT ("Animals"(Mesh) NOT "Humans"(Mesh))) AND English(Language)
Embase	('radiopharmaceutical agent'/exp OR 'radiopharmaceutical agent':ti,ab) AND ('kidney disease'/exp OR 'kidney disease':ti,ab OR 'kidney failure'/exp OR 'kidney failure':ti,ab OR 'renal impairment':ti,ab OR 'glomerulus filtration rate'/exp OR 'glomerular filtration rate':ti,ab OR 'estimated glomerular filtration rate':ti,ab OR 'metabolic clearance'/exp OR 'renal clearance':ti,ab) NOT ('animal'/exp NOT 'human'/exp) AND (english)/lim AND (embase)/lim NOT ((embase)/lim AND (medline)/lim)

### Study selection

All titles and abstracts were screened, and we retrieved the full text of potentially relevant studies. Two researchers (I.d.R. and N.S.) independently assessed the full text of each study for relevance. We included studies that met the following inclusion criteria: They described patients diagnosed or treated with a radiopharmaceutical and who suffered from CKD, they used a radiopharmaceutical that is (at least partly) cleared renally, and they made recommendations for an adequate dose in these patients or gave additional advice. Studies were excluded if they gave no relevant information on dosing or aspects related to dosing, addressed only the radiation safety of staff, described renal imaging, or were review articles.

### Assessment of methodological quality

Two researchers (I.d.R. and N.S.) independently assessed the methodological quality of the included observational studies using the Newcastle–Ottawa Scale (NOS): Quality Assessment Form for Cohort and Case–Control Studies (Ottawa Hospital Research Institute 2019). For each study, we scored nine items in three domains: selection, comparability, and exposure or outcome. Scores were added to create an aggregate score. The NOS scores were converted to ratings of ‘good’, ‘fair’, or ‘poor’ according to Agency for Healthcare Research and Quality standards (Singh et al. 2015). Studies of good quality were defined as those awarded 3–4 stars in the selection domain *and* 1–2 stars in the comparability domain *and* 2 stars in the exposure or outcome domain. Fair studies were defined as those awarded 2 stars in the selection domain *and* 1–2 stars in the comparability domain *and* 1–2 stars in the exposure or outcome domain. Poor-quality studies were defined as those awarded 0–1 stars in the selection domain *or* 0 in the comparability domain *or* 0 in the exposure or outcome domain. Where opinions on a score differed, we consulted a third reviewer (E.v.P.) to reach consensus.

### Data collection

For studies meeting the selection criteria, we extracted data using a standardised approach. Two researchers (I.d.R. and N.S.) independently extracted the following data: (1) author and journal, (2) year of publication, (3) study design, (4) name(s) of radiopharmaceutical(s) and the administered dose(s), (5) indication, (6) number of patients with CKD, (7) stage of CKD, (8) where applicable, the type and timing of dialysis after administration of the radiopharmaceutical, (9) recommendation(s) for adjustment of dose, (10) other advice on aspects such as adjustment of dialysis or scintigraphy, (11) reasons for dose adjustment, and (12) study limitations. We standardised the radiopharmaceuticals’ names according to the Anatomical Therapeutic Chemical classification system (WHO Collaborating Centre 2019), and the International Consensus Radiochemistry Nomenclature Guidelines (Coenen et al. 2019). Where studies reported the administered dose using the unit curie (Ci), we converted this to the SI derived unit becquerel (Bq) for uniformity of outcome (Allisy 1995). Patients with CKD were classified using the terminology of the CKD standard (Table 2), which includes five stages of kidney damage, from kidney damage with normal kidney function in stage 1 to kidney failure in stage 5 (National Kidney Foundation, Inc 2019; International Society of Nephrology 2013). We classified patients on dialysis as stage 5 if they had not been assigned to a specific stage in a study. When the extracted data were not in agreement and consensus could not be reached between the two researchers, a third researcher (E.v.P.) was consulted to resolve discrepancies.

**Table 2:** Classification of CKD by GFR (International Society of Nephrology^2013^; National Kidney Foundation, Inc 2019)

Stage	Description	GFR, mL/min/1.73 m^2^
1	Kidney damage with normal or increased GFR	≥ 90
2	Kidney damage with mild decreased GFR	60–89
3	Moderately decreased GFR	30–59
4	Severely decreased GFR	15–29
5	Kidney failure	< 15 (or dialysis)

Abbreviations: CKD, chronic kidney disease; GFR, glomerular filtration rate

## Results

### Search results

**Fig. 1:**
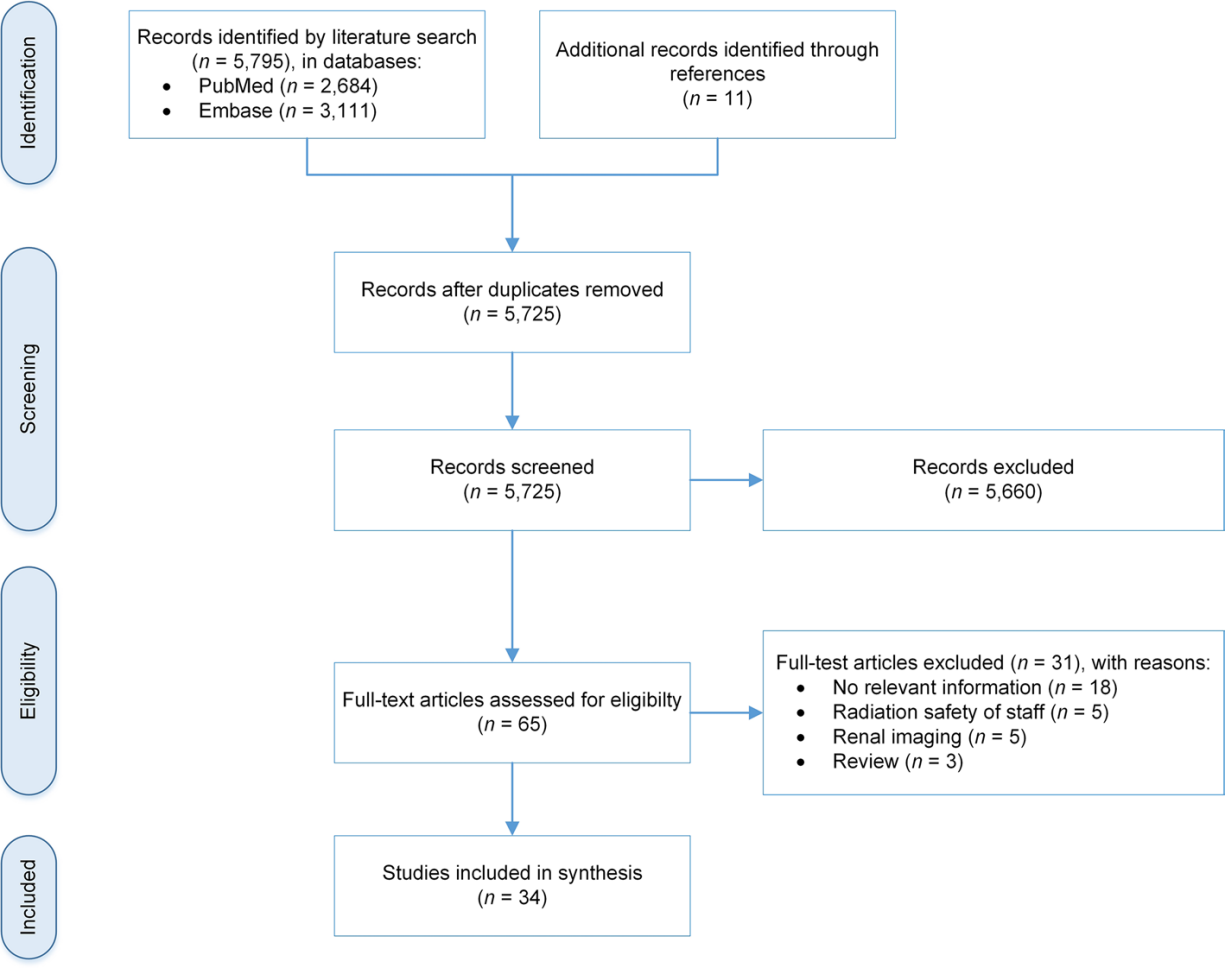
Selection of studies according to the PRISMA statement (Moher et al. 2009)

The literature search identified a total of 5795 studies in PubMed (*n* = 2,684) and Embase (*n* = 3111); another 11 studies were identified from references. After removing duplicates (*n* = 81), we screened 5725 studies by title (and abstract, where necessary), resulting in 65 potentially relevant studies. After a full-text screening we excluded another 31 studies for various reasons: they gave no relevant information on dosing or aspects related to dosing (*n* = 18), they addressed only the radiation safety of staff (*n* = 5), described renal imaging (*n* = 5), or were review (*n* = 3). A total of 34 studies remained for inclusion in this systematic review (Akers et al. 2016; Aktaş et al. 2008; Alevizaki et al. 2006; Bhat et al. 2017; Courbon et al. 1997,2006; Culpepper et al. 1992; Daumerie et al. 1996; Demko et al. 1998; Driedger et al. 2006; El-Zeftawy et al. 2017; Fofi et al. 2013; Holst et al. 2005; Howard and Glasser 1981; Jiménez et al. 2001; Kaptein et al. 2000; Kode et al. 2017; Laffon et al. 2008; Magné et al. 2002; McKay and Malaroda 2019; McKillop et al. 1985; Mello et al. 1994; Minamimoto et al. 2007; Miyasaka et al. 1997; Morrish et al. 1990; Pahlka and Sonnad 2006; Sinsakul and Ali 2004; Tobes et al. 1989; Toriihara et al. 2015; Toubert et al. 2001; Vermandel et al. 2020; Wang et al. 2003; Willegaignon et al. 2010; Yeyin et al. 2016). The selection process is illustrated in a PRISMA flow diagram (Fig. 1).

The 34 selected studies included 12 case reports (35.3%), 11 case series (32.4%), five case–control studies (14.7%), two cohort studies (5.9%), three theoretical models (8.8%), and one case report with a theoretical model (2.9%). The radiopharmaceuticals reported in these studies are [^18^F]fludeoxyglucose (FDG) (*n* = 5; 14.7%), [^131^I]sodium iodine (*n* = 28; 82.4%), and [^131^I]iobenguane (*n* = 1; 2.9%). Twenty-nine studies reported data for patients with CKD stage 5 (85.3%), while three studies included patients in other stages (8.8%). In two studies, the CKD stage was not identified, or it was determined in a non-standard fashion (5.9%). Patients in the 29 studies reporting data for CKD stage 5 were on renal replacement therapy. Nineteen studies described patients on haemodialysis (HD) (65.5%), four studies described patients on continuous ambulatory peritoneal dialysis (CAPD) (13.8%), three studies described patients on HD or CAPD (10.3%), one study described patients on HD or intermittent peritoneal dialysis (IPD) (3.4%), one study described patients on continuous haemodialysis (3.4%), and one study did not specify the type of dialysis (3.4%). In these studies, the timing of dialysis varied. For HD, the start of dialysis varied from 15 to 72 h after administration of the radiopharmaceutical, the number of dialyses varied from one to five times, and the timing intervals varied. For CAPD, the fluid changes varied from four to eight times a day. An overview of the included studies’ characteristics is presented in Table 3.

**Table 3:** Overview of included studies with their characteristics

References	Year	Study design	Number of patients	Radiopharmaceutical	Indication	Dose (MBq)	Stage of kidney failure (CKD)	Type of dialysis (number of patients)	Timing of dialysis after administration radiopharmaceutical
Akers et al. (2016)	2016	COS	58	[^18^F]fludeoxyglucose	PET/CT	370–555	1–5	NA	NA
Aktaş et al. (2008)	2008	CCS	10	[^131^I]sodium iodine	TC	1110–3700	5	HD (6); CAPD (4)	24 h continued every day for 5d; CAPD increased from 4 to 6–8 times a day
Alevizaki et al. (2006)	2006	CS	5	[^131^I]sodium iodine	PTC	1110–2590	5	HD (4); IPD (1)	48 h, and 2 patients also 96 h
Bhat et al. (2017)	2017	CR	1	[^131^I]sodium iodine	PTC	1850	5	HD	15 h, 27 h, 43 h
Courbon et al. (1997)	1997	CR	1	[^131^I]sodium iodine	TC	3700	5	HD	2d, 4d
Courbon et al. (2006)	2006	CS	2	[^131^I]sodium iodine	TC	3700	5	HD	72 h, 122 h-144 h
Culpepper et al. (1992)	1992	CR	1	[^131^I]sodium iodine	FTC	4773	5	HD	24 h,43 h,66 h
Daumerie et al. (1996)	1996	CS	3	[^131^I]sodium iodine	PTC	2 treatments of 925	5	HD	2d, 3d
Demko et al. (1998)	1998	CR	1	[^131^I]sodium iodine	TMNG	1045.62	5	HD	24 h
Driedger et al. (2006)	2006	CS	3	[^131^I]sodium iodine	PTC	3700; 3700; 2500	5	HD (2); CAPD (1)	NA
El-Zeftawy et al. (2017)	2017	CCS	27	[^131^I]sodium iodine	DTC	Mean dose 5550	3 and 4	NA	NA
Fofi et al. (2013)	2013	CS	2	[^131^I]sodium iodine	PTC	1850	5	CHD	24 h, 48 h
Holst et al. (2005)	2005	CR and TM	1	[^131^I]sodium iodine	PTC^a^	3637	5	HD	2d, 3d, 4d
Howard and Glasser (1981)	1981	CR	1	[^131^I]sodium iodine	PTC	740	5	HD	NA
Jiménez et al. (2001)	2001	CS	3	[^131^I]sodium iodine	PTC	2775; 3219; 4440	5	HD	24 h, 48 h, 72 h, 96 h, 144 h
Kaptein et al. (2000)	2000	CS	2	[^131^I]sodium iodine	PTC	980; 1110	5	CAPD	3–5 times a day
Kode et al. (2017)	2017	CCS	30	[^18^F]fludeoxyglucose	PET/CT	5.18 /kg	4 and 5	NA^b^	NA
Laffon et al. (2008)	2008	TM	NA	[^18^F]fludeoxyglucose	NA	NA	NA	NA	NA
Magné et al. (2002)	2002	CR	1	[^131^I]sodium iodine	PTC	1850	5	HD	24 h, 72 h, 144 h
McKay and Malaroda (2019)	2019	TM	NA	[^131^I]sodium iodine^c^	TD	NA	5	NA	Several timing intervals were simulated
McKillop et al. (1985)	1985	CR	1	[^131^I]sodium iodine	GD	462.5	5	HD	3d
Mello et al. (1994)	1994	CR	1	[^131^I]sodium iodine	PTC	2 treatments of 3700	5	HD	41 h, 89 h
Minamimoto et al. (2007)	2007	COS	20	[^18^F]fludeoxyglucose	PET/CT	210–360	NA^d^	NA	NA
Miyasaka et al. (1997)	1997	CR	1	[^131^I]sodium iodine	GD	740	5	HD	24 h
Morrish et al. (1990)	1990	CR	1	[^131^I]sodium iodine	PTC	1850; 4440; 5550; 9250	5	HD	48 h, 96 h, 144 h
Pahlka and Sonnad (2006)	2006	TM	NA	[^131^I]sodium iodine	TC	NA	5	HD; CAPD	Several timing intervals were simulated
Sinsakul and Ali (2004)	2004	CS	2	[^131^I]sodium iodine	PTC	3700; 5809	5	HD	20 h-24 h
Tobes et al. (1989)^e^	1989	CCS	1	[^131^I]iobenguane	PC	18.5	5	HD	NA
Toriihara et al. (2015)	2015	CCS	24	[^18^F]fludeoxyglucose	PET/CT	3.7 /kg	5	HD	NA
Toubert et al. (2001)	2001	CR	1	[^131^I]sodium iodine	FTC	814	5	CAPD	4 times a day
Vermandel et al. (2020)	2020	CS	6	[^131^I]sodium iodine	TC	1842–3747	5	HD	42 h, 90 h
Wang et al. (2003)	2003	CS	2	[^131^I]sodium iodine	PTC	3700; 5550	5	CAPD	4 times a day
Willegaignon et al. (2010)	2010	CR	1	[^131^I]sodium iodine	DTC	3700	5	CAPD	NA
Yeyin et al. (2016)	2016	CS	3	[^131^I]sodium iodine	DTC	2775 and 1850; 2775; 1850	5	HD	24 h, 48 h

CAPD, continuous ambulatory peritoneal dialysis; CCS, case–control study; CHD, continuous haemodialysis; CKD, chronic kidney diseases; COS, cohort study; CR, case report; CS, case series; DTC, differentiated thyroid cancer; FTC, follicular thyroid cancer; GD, Graves' disease; HD, haemodialysis; IPD, intermittent peritoneal dialysis; NA, not available; PC, pheochromocytoma; PET/CT, positron emission tomography/computed tomography; PTC, papillary thyroid cancer; TC, thyroid cancer; TD, thyroid disease; TM, Theoretical model; TMNG, toxic multinodular goiter

^a^Although the study describes hyperthyroidism as well, the reported case was treated with [^131^I]sodium iodine for thyroid cancer

^b^12 patients were on dialysis, type and timing were not specified

^c^In this study a pharmacokinetic software model was developed and validated with data of ^131^I, ^123^I and ^124^I

^d^Patients with a blood serum creatinine level > 1.1 mg/dl

^e^The findings in one patient were compared with two patients with mild to moderate renal function

### Quality of the observational studies

We performed a methodological quality assessment of the included observational studies, namely five case–control studies (Table 4) and two cohort studies (Table 5). Our assessment yielded one study rated ‘good’ (14.3%), one study rated ‘fair’ (14.3%), and five studies rated ‘poor’ (71.4%).

**Table 4:** Newcastle–Ottawa Scale assessment for case–control studies (Ottawa Hospital Research Institute 2019; Singh et al. 2015)

Study	Selection				Comparability		Exposure			Total score
	Adequate definition of case	Representativeness of cases	Selection of controls	Definition of controls	Comparability of cases and controls on the basis of the design or analysis	Study controls for any additional factor	Ascertainment of exposure	Same method of ascertainment for cases and controls	Non-response rate	
Aktaş et al. (2008)	0	0	0	0	0	0	0	*	0	1
El-Zeftawy et al. (2017)	0	*	0	0	*	*	0	*	0	4
Kode et al. (2017)	0	*	0	0	*	*	0	*	0	4
Tobes et al. (1989)	0	0	0	0	0	0	0	*	0	1
Toriihara et al. (2015)	0	*	0	*	*	*	0	*	0	5

'*' means one awarded point

Good-quality: awarded 3–4 stars in the selection domain and 1–2 stars in the comparability domain and 2 stars in the exposure domain. Fair studies: awarded 2 stars for selection and 1–2 stars for comparability and 1–2 stars for exposure. Poor-quality: awarded 0–1 stars for selection or 0 for comparability or 0 for exposure (Singh et al. 2015)

**Table 5:** Newcastle–Ottawa Scale assessment for cohort studies (Ottawa Hospital Research Institute 2019; Singh et al. 2015)

Study	Selection				Comparability		Outcome			Total score
	Representativeness of the exposed cohort	Selection of the non-exposed cohort	Ascertainment of exposure	Demonstration that outcome of interest was not present at start of study	Comparability of cohorts on the basis of the design or analysis	Study controls for any additional factor	Assessment of outcome	Follow-up long enough for outcomes to occur	Adequacy of follow-up of cohorts	
Akers et al. (2016)	*	*	0	0	0	0	0	*	*	4
Minamimoto et al. (2007)	*	*	0	*	*	0	0	*	*	6

'*' means one awarded point

Good-quality: awarded 3–4 stars in the selection domain and 1–2 stars in the comparability domain and 2 stars in the outcome domain. Fair studies: awarded 2 stars for selection and 1–2 stars for comparability and 1–2 stars for outcome. Poor-quality: awarded 0–1 stars for selection or 0 for comparability or 0 for outcome (Singh et al. 2015)

### Diagnostic radiopharmaceuticals and CKD

Regarding diagnostic radiopharmaceuticals in patients with CKD, we found dose recommendations and advice only for the radiopharmaceutical FDG (Table 6). The following section summarises our findings for this radiopharmaceutical.

**Table 6:** Overview of dose recommendations and other advice for diagnostic radiopharmaceuticals

Radiopharmaceutical	Stage of kidney failure (CKD)	Dose recommendation	Other advice
[^18^F]fludeoxyglucose	1, 2, 3, 4 or 5	No adjustment in dose or protocol is needed (Akers et al. 2016; Kode et al. 2017)	No adjustment in image time (Akers et al. 2016; Kode et al. 2017)
		Dose adjustment should be based on the optimized radiation dose (Laffon et al. 2008)	The more severe the kidney failure, the later the imaging should be, without necessarily beginning the acquisition beyond 160 min after injection (Laffon et al. 2008)
		ND	No large impact on assessment of scan (Minamimoto et al. 2007)
	5	ND	The effect of elevated FDG uptake in the background organs or blood pool may influence interpretation of the image in patients with CKD on haemodialysis (Toriihara et al. 2015)

CKD, chronic kidney diseases; FDG, [^18^F]fludeoxyglucose; ND, ‘not determined’

#### [^18^F]fludeoxyglucose (FDG)

A total of five studies reported the use of FDG in a total of 132 patients with CKD in several stages of CKD. Two of these studies recommended that no adjustment in dose or protocol is needed for patients with CKD. These studies based their recommendation on evidence that standardised uptake values in patients with CKD were comparable to those in patients with normal kidney function (Akers et al. 2016; Kode et al. 2017). One study suggested that a slight decrease in uptake in the brain and a slight increase in normal blood pool activity were caused by a higher FDG concentration in the blood and decreased uptake by tissues, and it concluded that these changes would not have a large impact on the assessment of the scan (Minamimoto et al. 2007). However, another study indicated that FDG uptake in background organs or blood pool might influence interpretation of the scan. In this case–control study, the standard uptake values (SUV)—normalised by body weight, as the control subjects had a greater body weight—in the gluteal muscles, subcutaneous fat, spleen, and right atrium were higher in patients on HD than in control subjects. The increased background uptake may influence quantitative measurements when the SUV of the background is used as a reference to assess tumour treatment response (Toriihara et al. 2015). A last study suggested, based on a theoretical assessment, to increase the time between radiopharmaceutical administration and imaging (Laffon et al. 2008).

### Therapeutic radiopharmaceuticals and CKD

For the use of therapeutic radiopharmaceuticals in patients with CKD, we found dose recommendations and other advice for the treatment of hyperthyroidism and thyroid cancer with [^131^I]sodium iodine and for the treatment of pheochromocytoma with [^131^I]iobenguane (Table 7). The following sections summarise findings for these treatments.

**Table 7:** Overview of dose recommendations and other advice for therapeutic radiopharmaceuticals

Radiopharmaceutical	Indication	Stage of kidney failure (CKD)	Dose recommendation	Other advice
[^131^I]sodium iodine	Hyperthyroidism	5	This study used a dose based on the 24-h radioiodine uptake and the weight of the gland (Demko et al. 1998)	Timing of dialysis should be consistent for both 24-h uptake study and treatment, and the most reasonable time for dialysis is 24 h after administration (Demko et al. 1998)
			This study used a dose of approximately one-third of the dose based on the 24-h radioiodine uptake and the weight of the gland (Miyasaka et al. 1997)	ND
			ND	Standard methods of management for hyperthyroidism are effective (McKillop et al. 1985)
	Thyroid cancer	3 and 4	Guidelines should consider adjusting the dose of [^131^I]sodium iodine to avoid possible harmful effects of excess [^131^I]sodium iodine on vital organs (El-Zeftawy et al. 2017)	ND
		5	Lower therapeutic doses are recommended (Aktaş et al. 2008; Pahlka and Sonnad 2006)	Reconsider alternate treatment (Aktaş et al. 2008)
				The dialysis frequency and the time interval between dose administration and dialysis can both be used effectively (Pahlka and Sonnad 2006)
			Lower therapeutic doses are recommended. The patient has to be administered around 75% of normal dose (Fofi et al. 2013; Vermandel et al. 2020)	Daily HD until a safe value of radioactivity for discharge was reached (Fofi et al. 2013)
				For metastatic patients pretherapeutic dosimetry studies are recommended (Vermandel et al. 2020)
			Lower therapeutic doses are recommended. The patient has to be administered around 50% of normal dose (Alevizaki et al. 2006; Bhat et al. 2017)	Administer the dose as soon as possible after dialysis, while the 48-h dialysis schedule of the patient could be carried on after [^131^I]sodium iodine treatment (Alevizaki et al. 2006)
				The dose recommendation is based on dialysis timing and frequency of the patient (Bhat et al. 2017)
			Lower therapeutic doses are recommended. The patient has to be administered around 25% of normal dose (Daumerie et al. 1996; Holst et al. 2005; Howard and Glasser 1981; Kaptein et al. 2000; Toubert et al. 2001)	First dialysis 24 h after radioiodine administration (Daumerie et al. 1996)
				Dialysis ideally should be performed just prior to the dose of [^131^I]sodium iodine. For radiation monitoring and precautions should be used for the first 3–4 dialysis sessions after treatment for thyroid cancer (Holst et al. 2005) Treatment requires multidisciplinary approach involving the endocrinologist, nuclear medicine physician, nephrologist, radiation safety team, and dialysis team (Holst et al. 2005)
				Arrange dialysis 48 h after the dose (Howard and Glasser 1981)
				Based on the calculations for CAPD (Kaptein et al. 2000)
			Higher therapeutic doses are recommended (Magné et al. 2002; Morrish et al. 1990)	Initiate the first dialysis after administration (Magné et al. 2002)
				Treatment procedure can be performed easily without significant radiation contamination or danger to personnel if proper precautions are observed (Magné et al. 2002)
				Delaying dialysis to 48 h (Morrish et al. 1990)
			Individual patient dosimetry/calculations are needed to make a dose recommendation (Courbon et al. 2006; Culpepper et al. 1992; Holst et al. 2005; Jiménez et al. 2001; Mello et al. 1994; Sinsakul and Ali 2004; Willegaignon et al. 2010; Yeyin et al. 2016)	Proper precautions for contamination of dialysis equipment and staff exposure should be taken (Courbon et al. 2006)
				If individual dosimetry is not available, administer 25% of the normal dose (Holst et al. 2005)
				Daily HD during the first 5 days of treatment (Jiménez et al. 2001)
				Discussions with personnel from the dialysis department, radiation safety and nuclear medicine are essential in planning and execution (Mello et al. 1994)
				Careful considerations regarding the timing of dialysis must be made (Sinsakul and Ali 2004)
				Considerations about safety must be made (Sinsakul and Ali 2004)
			ND	Dialysis could be done earlier to decrease the absorbed dose (Courbon et al. 1997)
				Stimulation with rhTSH simplifies the selection of ^131^I-doses in euthyroid dialysis patients (Driedger et al. 2006)
				Use of CAPD because of ease with which contamination with radiation could be prevented (Wang et al. 2003)
	Thyroid disease (not specified)		Factor of 3 × dose reduction (McKay and Malaroda 2019)	Optimum thyroid/reminder cumulated activity ratio for dialysis starting between 36 and 48 h (McKay and Malaroda 2019)
[^131^I]iobenguane	Pheochromocytoma	5	It may be prudent to reduce the administered dose of [^131^I]iobenguane given to CKD patients (Tobes et al. 1989)	The alteration in biodistribution in CKD must be considered in the interpretation of [^131^I]iobenguane scintigraphy and in the radiation dosimetry (Tobes et al. 1989)

CAPD, continuous ambulatory peritoneal dialysis; CKD, chronic kidney diseases; HD, haemodialysis; ND, ‘not determined’; rhTSH, recombinant human thyroid stimulating hormone

#### [^131^I]sodium iodine for the treatment of hyperthyroidism

Three case reports reported a total of three patients with CKD stage 5 on HD treated with [^131^I]sodium iodine for hyperthyroidism. Two of these studies calculated the dose of [^131^I]sodium iodine based on the 24-h radioiodine uptake and the weight of the thyroid gland (Demko et al. 1998; Miyasaka et al. 1997). In one of these two studies, HD was started after 24 h. Approximately one-third of the calculated dose was administered, although the reason for reducing the dose in this way was not given (Miyasaka et al. 1997). The other study emphasised the importance of consistency in the timing of dialysis for both the iodine uptake assessment and the treatment, and it stated that the most reasonable time for dialysis is 24 h after administration (Demko et al. 1998). The third case-report study did not specify dose calculations, but it stated that standard management for hyperthyroidism is effective (McKillop et al. 1985). Of the three patients in these case reports, one developed hypothyroidism three months after treatment and later reached a euthyroid state (McKillop et al. 1985), and two patients remained in a euthyroid state (Demko et al. 1998; Miyasaka et al. 1997). Exact follow-up times were not given for these three patients.

#### [^131^I]sodium iodine for the treatment of thyroid cancer

A total of 25 studies and case reports reported a total of 80 patients with CKD treated with [^131^I]sodium iodine for thyroid cancer. Of these studies, 24 (96.0%) included patients with CKD stage 5 who were on dialysis. Only one study included patients with CKD stages 3 and 4.

The latter study described the treatment of 27 patients with [^131^I]sodium iodine for ablation after thyroid cancer and reported a longer hospital stay and delayed renal clearance. It concluded that guidelines should consider adjusting the dose of [^131^I]sodium iodine in these patients to avoid increased radiation exposure (El-Zeftawy et al. 2017), but it did not specify the exact adjustment needed.

The dose recommendations differed in the studies with patients with CKD stage 5. Eleven studies (45.8%) recommended a lower therapeutic dose, whereas two studies (8.3%) recommended a higher therapeutic dose. Eight studies (33.3%) indicated that the therapeutic dose should be calculated by individual patient dosimetry, and three studies (12.5%) did not give dose recommendations but offered other advice. One study recommended that when it is not possible to calculate the therapeutic dose by individual patient dosimetry, a lower dose of 25% of the normal dose should be given (Holst et al. 2005). In the following paragraphs we describe the various dose recommendations for [^131^I]sodium iodine treatment of thyroid cancer in patients with CKD stage 5.

Two studies recommended a lower therapeutic dose in patients with CKD stage 5 but did not quantify the optimal dose (Aktaş et al. 2008; Pahlka and Sonnad 2006). One of these studies based this recommended dose on a theoretical pharmacokinetic model that included both CAPD and HD with several regimes starting after 24 h or 48 h (Pahlka and Sonnad 2006). The other study—performed in 10 patients, with four patients on CAPD and six patients on HD starting after 24 h—based their advice on higher and more persistent salivary gland, nasal, oral, and gastrointestinal uptake of [^131^I]sodium iodine in this group of patients. In this study, six patients experienced persistent xerostomia and one patient a transient epistaxis (Aktaş et al. 2008).

One study reduced the dose to 75% of the standard dose in two patients, based on literature also included in our review, and started HD 24 h after administration of the dose, followed by daily HD until a safe radiation dose rate was reached (Fofi et al. 2013). Another study recommended a 30% reduction in dose for ablative or adjuvant therapies and a dose based on pretherapeutic dosimetry studies for metastatic patients. This study based these recommendations on absorbed dose in the bone marrow estimated from normalised measured whole-body activity (Vermandel et al. 2020).

Two studies recommended a dose reduction of up to 50% of the dose given to individuals without CKD. In the first study, in five patients, the dose was reduced by 40% up to 50%, based on a previous case report and the researchers’ own experience. Dialysis in the patients in this study was started 48 h after treatment. The patients did not experience discomfort during hospitalisation, and four were reported to be free of recurrence after a follow-up period of three years. For one patient, treatment was too recent for a valid follow-up (Alevizaki et al. 2006). The second study described the successful treatment of one patient with a 50% reduction in dose based on maintaining a comparable area under the curve of a plot of ^131^I-iodine activity as function of time (Bhat et al. 2017).

Five studies recommended a dose reduction to 25% of a standard dose, but the type and timing of dialysis varied by study. One of these studies successfully treated three patients with this dose based on a blood activity concentration–time curve (Daumerie et al. 1996). The second study successfully treated a patient with a lower dose based on the literature, although this patient did experience mild transient sialadenitis, which is a known adverse effect (GE Healthcare 2020). The authors provided a mathematical analysis showing that a patient on HD receiving 21–28% of a normal dose in combination with dialysis on days 2, 3, and 4 receives the same dose as a patient with normal kidney function (Holst et al. 2005). The third study used a dose of 25% of the normal dose based on the measurement of blood activity of a small tracing dose of [^131^I]sodium iodine in a patient with HD and reported successful ablation of tumour remnants (Howard and Glasser 1981). The fourth study successfully used 22% of a normal dose in a patient on CAPD (Toubert et al. 2001). The last of these five studies reduced the doses given to two CAPD patients to 18%–20% of the dose given to a patient with normal kidney function. There was no recurrence of thyroid cancer in either patient after treatment after a follow-up period of seven to eight years (Kaptein et al. 2000).

Two studies recommended higher doses for dialysis patients than the doses used in patients with normal kidney function. Surprisingly, in one of these studies the authors suggested a dose of 125% of the normal dose, although they had treated a patient successfully with only 50% of a normal dose and started dialysis at 24 h. They based their recommendation for the higher dose on the shorter half-life of ^131^I calculated from dialysate samples (2.7 ± 0.8 h) in comparison with the half-life of ^131^I in a patient with normal kidney function (11.4 h) (Magné et al. 2002). The second study treated a patient with a higher dose than normal after an unsuccessful first treatment with a lower than normal dose (Morrish et al. 1990). The two studies disagreed on the timing of dialysis. One study recommended initiating dialysis right after [^131^I]sodium iodine administration (Magné et al. 2002), while the other study suggested delaying dialysis to 48 h after treatment to achieve a higher dose (Morrish et al. 1990). Another study indicated that the two studies recommending higher doses erred in their assumptions by not including a true effective half-life and not accounting for the almost complete lack of iodine clearance between dialysis sessions (Holst et al. 2005).

Eight studies did not give a dose recommendation but mentioned that the administered dose must be determined using individual patient dosimetry. Dosimetry prior to therapy aims to calculate the required dose by estimating the absorbable doses of radiation by internal organs and organs of interest based on the individual patient’s iodine kinetics using a low dose of ^131^I (Courbon et al. 2006; Culpepper et al. 1992; Holst et al. 2005; Jiménez et al. 2001; Mello et al. 1994; Sinsakul and Ali 2004; Willegaignon et al. 2010; Yeyin et al. 2016). These studies indicated that it is difficult to make a standard recommendation because of the large range in effective half-life and other variables between patients, such as differences in the amount of thyroid remnant or residual kidney function (Holst et al. 2005) and differences in dialysis protocols (Courbon et al. 2006). Based on a mathematical analysis, one study—also discussed in the section about studies recommending a dose reduction to 25% of a standard dose—stated that when individual calculations are not available, 25% of the normal dose should be administered (Holst et al. 2005). In addition to the recommendation to use individual dosimetry calculations, some studies also offered safety advice, such as stating proper precautions against contamination of dialysis and staff exposure (Courbon et al. 2006; Sinsakul and Ali 2004). One study emphasised that discussions between personnel from the dialysis department, radiation safety, and nuclear medicine are essential in planning and executing the treatment with [^131^I]sodium iodine (Mello et al. 1994). Three studies did not make dose recommendations but offered other recommendations (Table 7). One study described a theoretical model of [^131^I]sodium iodine dosing in thyroid disease but did not specify which disease (McKay and Malaroda 2019).

#### [^131^I]iobenguane

One case report reported the use of [^131^I]iobenguane in a patient with kidney failure and compared the findings with data from two patients with mild to moderate loss of kidney function. This study did not make a specific dose recommendation, but it did indicate that the administered dose of [^131^I]iobenguane in patients with CKD should be reduced, given their reduced renal clearance of [^131^I]iobenguane (Tobes et al. 1989).

## Discussion

Based on a systematic review of the literature, which included 34 studies, consistent recommendations about radiopharmaceutical dosing in patients with CKD cannot be given. While studies do mention difficulties with the dosing of these medicines in patients with CKD, information is available for only a few radiopharmaceuticals, and recommendations are often contradictory.

Results for the diagnostic radiopharmaceutical FDG suggest that adjustment of the dose is not required, but some effect on the uptake of FDG must be considered in interpreting the scan. We found no results for other diagnostic radiopharmaceuticals. This finding was unexpected and may suggest that, even though altered biodistribution due to CKD may lead to a poor target-to-non-target ratio with diagnostic radiopharmaceuticals, no significant influence on image quality is apparent in daily practice. We hypothesise that for most diagnostic radiopharmaceuticals, CKD may be less important because inadequate tissue distribution and background clearance do not lead to clinically significant issues published, and technical and patient-related factors have a more important influence on scan quality. Further work is needed to investigate the effect of CKD on the biodistribution of diagnostical radiopharmaceuticals and the diagnostic outcome.

For the therapeutic radiopharmaceutical [^131^I]sodium iodine, the dosing recommendations are not in agreement. For treatment of thyroid cancer, most studies recommend dosing based on individual patient dosimetry calculations. Other studies recommend changing the dose, with advice varying from lowering the dose by 75% to increasing the dose. The variations in dose recommendations for [^131^I]sodium iodine might be explained by variability in individual patients and in used methods, including (a) differences in the amount of remnant thyroid tissue and tumour stage in general; (b) variation in residual kidney function; (c) variability in effective half-life; and (d) differences in method, timing, and frequency of dialysis treatment (Alevizaki et al. 2006; Bhat et al. 2017; Pahlka and Sonnad 2006; Sinsakul and Ali 2004; Meller et al. 2002).

Although the findings for therapeutic radiopharmaceuticals were limited, some studies indicated that dose adjustments in patients with CKD are important, and that altered biodistribution does affect therapeutic response and the risk of toxicity for non-target organs. A non-optimised dose in patients with CKD may lead to inadequate treatment (Aktaş et al. 2008), increased radiation exposure (El-Zeftawy et al. 2017), or an increased risk of adverse effects (for example, bone marrow toxicity, xerostomia, epistaxis, sialadenitis, or xerostomia from treatment with [^131^I]sodium iodine) (Aktaş et al. 2008; Alevizaki et al. 2006; Holst et al. 2005; Vermandel et al. 2020).

We found studies for only two therapeutic radiopharmaceuticals, and none providing dose recommendations for radiopharmaceuticals such as [^177^Lu]Lu-oxodotreotide or [^90^Y]Y-octreotide, even though CKD is described as a risk factor in ^177^Lu-somatostatin analogue treatment (Bodei et al. 2008b; Svensson et al. 2012), and the radiopharmaceutical’s Summary of Product Characteristics warns that special consideration regarding the dose is required in these patients (Advanced Accelerator Applications 2020). In this regard, the European Medicines Agency has indicated that safety and efficacy studies often exclude patients with CKD (European Medicines Agency 2015). Therefore, we recommend including patients with CKD in studies, listing pharmacokinetic information regarding the influence of decreased kidney function, and providing specific dosing recommendations for therapeutic radiopharmaceuticals. With the introduction of new therapeutic radiopharmaceuticals, there is increased interest in patient-specific dosimetry. Although the practice of patient-specific dosimetry for therapeutic radiopharmaceuticals has been shown to vary across Europe (Sjögreen Gleisner et al. 2017), efforts are underway to optimise and standardise this practice (Stokke et al. 2017; Konijnenberg et al. 2021). Patient-specific dosimetry could play a role in predicting or verifying the dose of therapeutic radiopharmaceuticals in patients with CKD, and we suggest including CKD in future work on this topic.

Another finding is that most studies describe patients with CKD stage 5; only a few studies (8.8%) include patients in other CKD stages. As the estimated prevalence of CKD is 11–13% in the general population, with the majority in CKD stage 3 and only 0.1% in CKD stage 5, one would expect more information to be available on patients in CKD stages 1–4 (Hill et al. 2016). It is difficult to explain these results. Possible explanations are that the effects of CKD stages 1–4 on the results of the nuclear medicine examination are less pronounced than those of CKD stage 5, that information on kidney function in patients in lower CKD stages is not available to the nuclear medicine staff, or that to date, kidney function has not been considered when planning for a patient. To develop a full picture of the dosing of radiopharmaceuticals in all CKD stages, future studies should include not only patients with CKD stage 5, but also those in other stages.

Regarding patients in CKD stage 5 on renal treatment therapy, studies indicate that dialysis complicates the dosing of the radiopharmaceutical. Clearance of the radiopharmaceutical during dialysis may be altered or influenced by many aspects. First, characteristics of the substance such as molecular weight, solubility, binding to proteins, and volume of distribution are important to consider. In addition, dialysis-specific aspects such as the characteristics of the dialysis membrane, transmembrane pressure, dialysate flow rate, and timing and frequency of dialysis impact the clearance of the pharmaceutical (Velenosi and Urquhart 2014). These aspects complicate the treatment of patients with a therapeutic radiopharmaceutical and necessitate careful consideration of the timing and frequency of dialysis (Pahlka and Sonnad 2006; Sinsakul and Ali 2004). Other challenges for patients on renal treatment therapy receiving a therapeutic radiopharmaceutical include contamination of dialysis equipment and radiation exposure to staff. However, studies have shown that with proper precautions in planning and execution, the procedure can be performed safely (Courbon et al. 2006; Magné et al. 2002; Mello et al. 1994; Sinsakul and Ali 2004). A multidisciplinary approach, including the nuclear medicine physician, endocrinologist, nephrologist, radiation safety team, and dialysis team, is advised for treatment (Holst et al. 2005).

We believe our systematic review identifies an important gap in research for the dosing of radiopharmaceuticals in patients with CKD. The strength of our review is that we used a systematic approach and formulated a well-defined search string to identify available studies on radiopharmaceuticals and CKD. The researchers screened the included studies independently, which decreased the possibility of bias in this review.

A limitation of our review is that we only included published information on the dosing of radiopharmaceuticals in patients with CKD. Other data, such as data on file available from marketing authorisation holders or studies published in another language, may provide more insight into this subject. Although outside the scope of this study, an evaluation by an expert panel with diverse areas of expertise may also aid in compiling recommendations. Forming such an expert opinion is described for the safety evaluation of other drugs in combination with disease conditions and may well be a next step in clarifying the dosing of radiopharmaceuticals in patients with CKD (Weersink et al. 2018).

The quality of the included studies is not adequate for making reliable radiopharmaceutical dosing recommendations for patients with CKD. Most of the observational studies (71.4%) were determined to be of ‘poor’ quality using the NOS. The study that was determined to be of ‘good’ quality included aspects such as a well-defined selection of both cohorts (patients with CKD and healthy volunteers) and an adequate follow-up (Minamimoto et al. 2007). For many included studies (79.4%), a quality assessment using the NOS was not possible, because they were case reports and theoretical models. The quality of evidence is low, as many studies report only one case (such as dialysis patients) or consider only one factor (such as one radiopharmaceutical). It is apparent that more well-designed research needs to be done to include all radiopharmaceuticals and to include patients with CKD in various stages.

Uniform dose recommendations for radiopharmaceuticals based on kidney function are difficult to provide. Therefore, we recommend that additional research should be conducted to address this absence of information about radiopharmaceuticals and CKD and to provide appropriate dose recommendations for clinical practice. Evaluation of therapeutic radiopharmaceuticals in particular is important, because non-optimised dose for these radiopharmaceuticals may lead to inadequate treatment, increased radiation exposure, or an increased risk of adverse effects. With the introduction of new therapeutic radiopharmaceuticals, such as those based on alpha-emitting radionuclides, including patients with CKD may become even more important (Langbein et al. 2019).

## Conclusion

This study has determined that information on the dosing of radiopharmaceuticals in patients with CKD is limited. While some studies do mention difficulties with dosing these medicines in these patients, information is available for only a few radiopharmaceuticals and for only some CKD stages. Moreover, recommendations are sometimes contradictory. Further research on the dosing of radiopharmaceuticals in patients with CKD is needed to determine whether specific dosing is required, especially for therapeutic radiopharmaceuticals where a non-optimised dose may lead to an increased risk of toxicity for non-targeted organs. Including patients with CKD in studies and providing specific information about dosing in these patients should be a priority for the radiopharmaceutical community.

## Data Availability

The data that support the findings of this study are available from the corresponding author upon reasonable request.
